# Nutrition Patterns of Polish Esports Players

**DOI:** 10.3390/nu15010149

**Published:** 2022-12-28

**Authors:** Monika Szot, Barbara Frączek, Florentyna Tyrała

**Affiliations:** Department of Sports Dietetics, Gdansk University of Physical Education and Sport, 80-336 Gdansk, Poland

**Keywords:** esports, cognitive performance, video gaming, dietary patters, eating habits, cognitive performance, western diet

## Abstract

The aim of this study was to quantify the dietary patterns (DPs) of Polish esports players aged 18–26 years. Data were obtained via questionnaires that assessed dietary habits and frequency of food consumption. Dietary patterns were derived using Principal Component Analysis (PCA) and Factor Analysis (FA). In total, nine distinct DPs were identified. Eight DPs were considered unhealthy; ‘fast food’, ‘High-processed food, meat and confectionery’, ‘Sweet’, ‘Fat-diary products’, ‘Vegetable-fruit’, ‘Spices and additives’, ‘Fats’ and ‘Cereal’; and only one was deemed healthy. E-athletes presented mostly poor dietary habits, which included: irregular eating of meals, frequent snacking, at least three meals a day and composition of snacks, frying of meat dishes and sweetening of hot drinks. Healthy dietary habits included proper hydration during the day and consumption of mainly non-sparkling water. The unbalanced and largely unhealthy dietary habits of esports players raise health concerns for these e-athletes, particularly when combined with a sedentary lifestyle. Future research could assess the nutritional knowledge of this group as it relates to national guidelines, investigate interventions designed to introduce healthier eating options into their lifestyle and examine the relationship between DPs and health or cognitive performance.

## 1. Introduction

Electronic sports (esports) have gained high popularity and success in the global entertainment industry [1,2]. Esports represents organized and competitive video gaming, where individuals or teams of players compete against each other through human-computer interactions [3,4]. Esports requires a combination of motor, cognitive, strategic, and mimetic skills [5]. Several factors, such as proper diet, sleep pattern and fitness level, seem to be crucial for cognitive performance [6]. However, few studies have examined the health-related behaviors of esports players [7,8]. These players paid less attention to a healthy diet and were characterized by lower consumption of vegetables and fruits compared to daily recommendations for the general population [7]. It is clear that prolonged gaming is linked to poor dietary habits [9]. In general, DPs focus on the impact of the whole diet rather than examining individual nutrients and products within that diet, which could provide a stronger format to understand the complex relationship between diet and health [10,11]. Despite many studies of DPs among different populations, the food intake and diet quality of esports players have not been studied. Therefore, the purpose of this study was to describe the DPs of esports players in terms of the frequency of consumption of products that potentially affect brain function.

## 2. Materials and Methods

### 2.1. Participants and Survey Tools

This study was conducted in a group of 233 male esports players, 18–26 years (mean age was 20.5 ± 2.0), from 2019 to 2021. The cohort was considered well-trained, which we defined as receiving gratification and training under the supervision of a trainer (n = 47; 20.2%), and some were semi-professional players (n = 186; 79.8%). The participants’ average body mass was 76.3 ± 15.8 kg, and their height was 180.3 ± 6.3 cm. The inclusion criteria were: men, age ≥ 17 years, training and playing in esports for at least 3 years. The players represented diverse genres of games, such as Counter Strike: Global Offensive (CS:GO) (69.1%) and League of Legends (LOL) (30.9%). The average playing time was 4–6 h per day. Study data were obtained via self-administered, anonymous dietary questionnaires assessing dietary habits and frequency of food consumption. Additional socio-demographic information was collected by surveys, such as gender, age, weight, height, sport status, training experience and esports games. The study focus was on the assessment of dietary habits, i.e., the number and regularity of meals consumed, the frequency of eating meals, the composition of snacks eaten, the type of milk and milk products consumed, the type of culinary techniques used to prepare meat dishes, the type of fat used to spread and fry meals, sweetening of hot drinks and salting of food, the type of water consumed during the day, last meal practices and daily fluid consumption used the KomPAN questionnaire (part A) [12].

To assess the food consumption frequency of participants, we implemented a modified questionnaire based on the International Food Frequency Questionnaires (FFQ), Harvard Food Frequency [13] and National Health and Nutrition Examination Survey (NHANES) [14]. The FFQ was translated from English into Polish and adapted to reflect the Polish diet by adding the usual foods consumed in Poland to each section. In order to retain international comparability, part of the food items was kept. The adaptations were related to products, which potentially impact positively or negatively on brain function. Each food item in FFQ had assigned a portion size as grams or home measures (using a detailed table of home measures and grams of foods and selected food). The frequency of consumption scale was increased from 1 to 7, and portion sizes (e.g., average portion) were specified in each product. The FFQ consisted of 145 food items consumed during meals and between meals, at home and outside. It was used to assess the dietary intake of participants over the previous year. Briefly, the questionnaire contained 15 categories of foods and beverages: unsweetened drinks and soups (7 items); vegetables and vegetable preserves including vegetable juices (25 items); fruit and fruit preserves including fruit juices (17 items); legumes and legumes preserves (7 items); cereal products, potatoes, flour and potato dishes (14 items); milk and dairy products (13 items); meat and meat products (10); fishes and seafood (11 items); eggs (1 item); vegetable fats, nuts and seeds (21 items); animal fats (2); sugar and confectionery (2); other drinks—sweetened, energizing, alcohol (8 items); spices (5 items) and fast food (2 items). The food items in the FFQ were categorized into 37 food groups. The FFQ uses a seven-point scale of frequency of consumption, as follows: never/less than once a month (1), 1–3 times a month (2), once/twice a week (3), a few times a week (4), once a day (5), 2–3 times a day (6) and 4–5 times a day (7). For each food and beverage item, participants were asked to quantify their frequency of consumption in an open-ended format (using one of the seven options). To interpret the average values of the frequency of consumption, the following rankings were used: never/less than once a month (1–1.49), 1–3 times a month (1.5–2.49), once/twice a week (2.5–3.49), a few times a week (3.5–4.49), once a day (4.5–5.49), 2–3 times a day (5.5–6.49) and 4–5 times a day (6.5–7.00).

### 2.2. Derivation of Dietary Patterns

**Figure A1:**
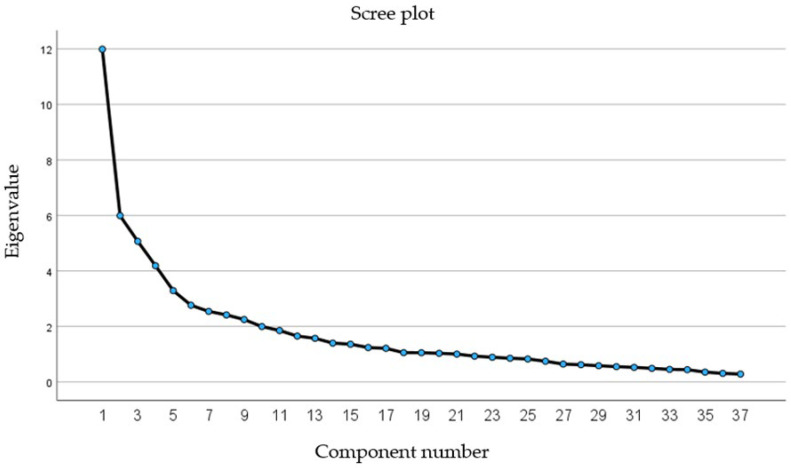
PCA scree plot.

DPs were empirically extracted using factor analysis (FA) of principal components (PCA), which is a multivariate statistical procedure. The 145 items from the FFQ were aggregated into 37 mutually exclusive food groups and individual food items. The frequency of consumption of the 37 food groups constituted the output factors of the variable base. Where appropriate, the Kaiser–Meyer–Olkin (KMO) test (to verify method applicability) and Bartlett’s test of sphericity (to test the null hypothesis of no relationship between variables) were carried out [15,16]. The KMO value was 0.82, and the significance of Bartlett’s Sphericity was below 0.001, indicating that FA was appropriate to use. Varimax orthogonal rotation was performed to maintain independent factors while improving interpretability. We identified the main factors (i.e., DPs) according to the eigenvalue (>1), a scree plot (Figure A1), factor interpretability and the proportion of variance explained by each factor [17]. The first 11 factors were identified, each accounting for at least 61.27% of the model variance. Food items with absolute factor loadings ≥0.40 were considered representative of each DP. Indeed, a 0.40 cut-off implied a minimum contribution of any factor to any food group’s total variance. Ultimately, 9 predominant factors constituting the DPs were subject to further analysis. Interpretation of the remaining factors was abandoned because they were made up of individual products. The total contribution of the dietary profiles in explaining model variance was 54.97%. The higher the values of the factor loadings, the stronger the association between a participant’s diet and the DP. The DPs were named according to the magnitude of the factor scores, published studies, cultural aspects and interpretability of the overall diet.

### 2.3. Statistical and Data Analyses

The statistical analyses were carried out using IBM SPSS 21^®^ software (IBM Corp., Amonk, NY, USA). Descriptive statistics were computed for all questions. Data are presented as numbers (n) and percentages (%) for qualitative data (categorical, nominal), whereas quantitative data are presented as means (M) and standard deviations (SD).

## 3. Results

### 3.1. Dietary Habits of the Study Subjects

A majority of the esports players had at least 3 meals per day (40.87%), followed by 45.21% who had greater than 3 meals per day. At the same time, 39.13% of respondents ate between meals at least once a day. The survey results demonstrated that only 10.43% of the sample ingested regular meals. A minority of esports players (31.30%) consumed their last meal 2–3 h before going to bed, but nearly 68.66% did not follow this recommendation. In terms of the type of snacks consumed, nearly 41.74% of respondents used fruit, but only every tenth esports player ate vegetables, and 12.17% of the sample consumed different types of nuts and oil seeds. On average, every third participant reached for sweets (39.13%) and salted snacks (28.70%). One in three participants reported using quick-cooking products five or more times a month, but almost 25% do not consume them at all. Consumption of milk and dairy products with standard fat content was declared by a higher percentage of esports players (66.09%) than those using products with a reduced fat content 27.83%). Regarding the less healthy culinary techniques, most of the respondents reported preparing meat dishes by frying (80.87%), followed by baking (53.04%) and boiling (45.22%). Only every third of the participants chose to grill to prepare meat dishes. Usually, the e-athletes used mainly vegetable fats (47.83%) for frying meals. The majority of respondents (56.90%) declared using butter to prepare sandwiches. Every second contestant claimed to sweeten hot drinks such as coffee, tea and cocoa with at least two teaspoons of sugar. A similar percentage of esports players indicated occasional salting of dishes and sandwiches (47.83%), but some did not follow this custom 40.00%). It was found that the majority of e-athletes (62.61%) drank at least 1.5–2 L of fluids a day, with a preference for non-sparkling water (69.57%) than sparkling water or fluids with additives. More detailed data are included in Table A1 of Appendix A.

### 3.2. Food Frequency Questionnaires

Table 1 presents the results of the frequency of food consumption that arguably, have a positive influence on brain function. The highest frequency of consumption was indicated for mineral water (5.56 ± 1.65), which was usually taken at least 2–3 times a day by nearly 2/3 of the respondents (Table A2). This food item was followed by hot beverages, spices, table and spring water, fruits, vegetables, milk, white meat dishes and eggs, usually consumed several times a week or more frequently by most of the esports players (55.36–71.67%). At the same time, vegetables were consumed at least 2–3 times a day by only 9.87% of e-athlete and milk (including flavored milks, cocoa, and coffee with milk) was consumed by 12.45%. Potatoes, olive oil and rapeseed oils, fruit juices, fermented milk drinks, buckwheats, other whole grains, cereals, whole grain pasta and whole grain bread were ingested once or twice a week. Less than a third of the respondents used olive oil or rapeseed oil as a cooking additive several times a week (3.33 ± 1.43). Only every tenth respondent chose yogurts and kefirs at least once a day, while 1/4 of the sample consumed these products exactly 1–2 times a week or several times a week. An equally low percentage of people observed the consumption of wholegrain bread—only 10% of respondents chose this product at least once a day (2.59 ± 1.53). Most, as many as 1/3 of the sample, did not include it in their diet or used it less frequently than once a month. Meanwhile, only 1/3 of the group consumed buckwheats, other whole grains, cereals and whole grain pasta at least several times a week (2.87 ± 1.24).

The least frequently consumed products (1–3 times a month) were cottage cheese, fish, nuts, pumpkin seeds, sunflower seeds and other oil seeds, legumes or dishes from legumes, vegetable or vegetable-fruit juices and vegetable canned, marinated or pickled vegetables (Table 1). Only 7.30% of respondents used nuts and oilseeds at least once a day, and the vast majority of esports players (59.23%) consumed them less than 1–3 times a month (Table A2). The participants rarely, on average 1–3 times a month, chose fish to eat (2.83 ± 0.93), and less than half of the sample (43.78%) consumed them 1–2 times a week or several times a week. Legumes (2.12 ± 1.13) were included in the diets of most competitors (67.81%), never or rarely, i.e., 1–3 times a month or less (Table 1, Table A2).

Table 2 presents the results concerning the frequency of consumption of products with an adverse effect on brain function. The participants most often consumed light bread, wheat, rye, mixed wheat and rye bread, toast, rolls and rogals (4.85 ± 1.29), with a lower frequency of consumption for margarine or mixes (2.27 ± 1.54), lard/coconut oil (2.17 ± 1.42), alcohol drinks (1.99 ± 1.03), powder soups or ready soups (1.88 ± 1.10) and meat canners (1.52 ± 0.95). The survey revealed that as many as 66.52% of respondents ate processed cereal products once a day or more. Other food products, such as fried food, meats and pork sausages, cheese, white rice, standard pasta and fine groats and sweets, were taken on average several times a week by most competitors (54.94–75.54%) (Table A3).

Less frequently (i.e., 1–2 times a week), the respondents drank sweet carbonated or non-carbonated beverages (3.39 ± 1.56). Moreover, every fourth e-athletes used these products several times a week. Also, a similar frequency of consumption was noted for fast foods (2.88 ± 1.07), red meat dishes (2.73 ± 1.24) and energy drinks (2.69 ± 1.44), which were consumed at least 1–2 times a week by less than 50% of respondents. Interestingly, every fourth e-gamer never or very rarely consumed energizing drinks (Table 2, Table A3).

### 3.3. Dietary Pattern Characterization

Nine DPs were identified and labeled: pattern one—‘Almost healthy’, pattern two—‘Western fast food’, pattern three—‘High-processed food, meat and confectionery’, pattern four—‘Sweet’, pattern five—‘Fat-diary products’, pattern six—‘Vegetable-fruit’, pattern seven—‘Spices and additives’, pattern eight—‘Fats’ and pattern nine—‘Cereal’. These patterns explained the total variance of 9.47%, 9.22%, 7.14%, 5.81%, 5.56%, 5.34%, 4.61%, 4.08% and 3.73% respectively (Table 3). Pattern one: ‘Almost healthy’ was characterized by white rice, standard pasta, fine groats (manna, cuscus), whole grain bread, white meat dishes (chicken, turkey, rabbit), vegetables, and eggs which were eaten several times a week on average, fishes, nuts, pumpkin seeds, sunflower seeds and other oil seeds, legumes or dishes from legume seeds (beans, peas, lens, soya) and cottage cheese (homogenized cheese, cheese deserts) which were eaten 1–3 times a month. Pattern two: ‘Western fast food/Unhealthy’ included fast food (fries, hamburgers, pizza, chips) consumed on average 1–2 times a week, legumes or dishes from legume seeds (beans, peas, lens, soya), canned meat, vegetable canned, marinated or pickled vegetable, vegetable or vegetable-fruit juices, powder soups or ready soups (can, jar, concentrated) and alcohol drinks eaten less frequently, i.e., 1–3 times a month on average. Pattern three: ‘High-processed food, meat and confectionery’ was characterized by intake of light bread (wheat, rye, mixed wheat and rye bread, toast, rolls, rogals) consumed at least once a day, meats, pork sausages, fried food (meat or flour) and sweets (cookies, cakes, chocolate bars, ‘muesli’ types and other confectionery products, consumed on average several times a week and meat, sausage, poultry and veal eaten on average 1–2 times a week. Pattern four: ‘Sweet’ included sweets (cookies, cakes, chocolate bars, ‘muesli’ types and other confectionery products) consumed on average several times a week and sweet carbonated or non-carbonated beverages (Coca-cola, Pepsi, Sprite, Fanta, lemonade), energy drinks (red bull, shot, black horse and others) which were eaten less frequently, i.e., 1–2 times a week. Pattern five: ‘Fatty-diary products’ was characterized by intake of cheese (molded cheese, blue cheese) on average several times a week, fermented milk drinks (yogurt, kefir) consumed 1–2 times a week, and cottage cheese (homogenized cheese, cheese deserts) which were consumed less often, i.e., 1–3 times a month. Pattern six: ‘Vegetable-fruit’ included vegetables, fruits and hot beverages (tea, coffee, herb or fruit infusions), which were eaten on average several times a week, and fruit juices, consumed 1–2 times a week. Pattern 7: ‘Spices and food additives/oils’ was characterized by intake of spices, which were consumed on average several times a week, and olive oil and rapeseed oils eaten less often, i.e., 1–2 times a week. Pattern eight: ‘Fats’ included margarine or mixes with butter and margarine and lard/coconut oil, rarely consumed 1–3 times a month on average. Pattern nine: ‘Cereal’ was characterized by a higher consumption of buckwheats, other whole grains, cereals, whole grain pasta and a lower consumption of lard and coconut oil.

## 4. Discussion

To our knowledge, this study is the first attempt to identify and characterize DPs among Polish professional and semi-professional esports players, taking into account potential beneficial and detrimental products for brain health. There is sufficient evidence that has investigated the relationship between specific nutrients and brain function [18,19,20]. Based on the obtained data from previous studies, we have divided products with beneficial and adverse effects on brain function (Table 1 and Table 2). Using factors analysis, the main nine DPs were identified. Among them, eight were unhealthy, and one was considered as healthy.

High cognitive abilities (intelligence, perception, spatial orientation, attention, and dual-tasking) are required for many esports games, such as League of Legends and Counter-Strike: Global Offensive [21,22]. So far, a limited number of studies have focused on describing the cognition abilities of esports players [23,24,25].

Defining a healthy DP is important for creating dietary guidelines and defining practices to monitor and improve diet quality, both at the individual level and for the general population [26]. Adherence to the Mediterranean diet (MeDi) pattern in middle-aged and elderly populations correlates with better cognitive abilities [27,28,29]. Our list (Table 1) is consistent with the main characteristics of MeDi, including consumption of fruits, vegetables, olive oils, whole grains products, fish, low-fat milk products, poultry, tea, coffee and reduced consumption of red meat, sausages and refined cereals, which negatively impact on brain function (Table 2) [30].

An ‘Almost healthy’ DP is characterized by high-frequency consumption of white meat and vegetables, which are potentially associated with better brain function (Table 1). Other foods in this pattern, such as white rice, standard pasta and fine groats, were consumed less frequently, and their possible impact on brain function was identified as being negative (Table 2).

‘Western food’ mainly consists of fast-food products such as fried chips, hamburgers, pizzas and potato chips, which are part of the Western diet (WD). Western DPs consist of a high intake of fats, carbohydrates, processed foods and red meats (as a source of protein) with a reduced intake of fruits and vegetables [31,32]. In previous studies among young adults, western patterns have also been identified [33,34,35,36,37]. Long-term adherence to habitual consumption of WD contributes to the development of metabolic diseases and psychological problems [38,39]. A growing body of evidence has demonstrated associations between Western DP and cognitive function [40,41,42]. Altering the composition of gut microbiota and interactions among the brain-axis can negatively affect brain function [38]. Experimental studies conducted on animals, and a few on humans, have shown that the excessive intake of WD can lead to hippocampal dysfunction and, consequently, to cognitive impairments [42,43].

The next two identified DPs also represent the westernized diet. ‘High-processed food, meat and confectionery’ DP is characterized by frequent high consumption of light bread and less frequently consumed fried food, meats, pork and sweets. This pattern resembles traditional Polish cuisine, with white flour products, fried foods, fatty meats and sweets [44,45]. Frequent ingestion of the above-mentioned products is detrimental to health and brain functions (Table 2). ‘Sweet’ DP is based on a high-frequency intake of sweets and less frequent consumption of sweet carbonated, non-carbonated beverages and energy drinks. ‘Fatty-dairy products’ DP included a high intake of different types of cheese and other dairy products, which were consumed less often. Some findings suggest that there are associations between frequent dairy foods intakes with better cognition [46]. One recent report of a study involving esports players aged 16–36 years found no link between dairy intake and cognitive performance [47].

‘Vegetable-fruit’ DP represents a healthy DP, consisting of a high frequency of vegetables, fruits and hot beverages (tea, coffee, herb) or fruit infusions, although fruit juices are consumed with lower frequency. Fruits and beverages such as tea and coffee are the main sources of dietary polyphenols, which have a positive effect on cognition and memory [19,48,49]. However, one study found that habitual coffee and tea drinking had mixed effects on cognitive improvement [50]. Scientific evidence confirmed that adequate vegetable and fruit consumption has a beneficial influence on health and can prevent cognitive impairment due to the presence of antioxidants and anti-inflammatory factors [51,52].

The positive cognitive effects of bioactive compounds also depend, in part, on portion sizes and frequency of consumption [53]. Essaw et al. concluded that young undergraduate students do not consume fruits and vegetables every day due to a higher cost compared to other foods [54]. ‘Spices and food additives’ DP are characterized by the intake of spices on average several times a week and less consumption of olive oil and rapeseed oils. There are findings that the effects of herbs and plant ingredients may positively affect cognitive performance and memory, but there is a need for further research [19,55].

Some evidence supports the idea that the consumption of extra-virgin olive oils can promote brain health and prevent cognitive decline [56]. ‘Fats’ DP consisted of frequent intake of margarine or mixes with butter and margarine and lard with rarely consumed coconut oil. A diet rich in trans isomers (TFAs) and saturated fatty acids (SFAs) may carry a risk of cognitive disorders [57]. In contrast, the consumption of omega-3 fatty acids, which are components of cell membranes, can promote normal cerebral functions and cognitive abilities [17,58]. Omega-3 fatty acids could prevent cognitive decline, but confirmatory evidence is scarce [59]. Sánchez-Villegas et al. indicated the relationship between TFAs consumption and depression risk among Spanish university graduates, while a weak inverse was noted for monounsaturated fatty acids (MUFAs), polyunsaturated fatty acids (PUFAs) and olive oil [60]. ‘Cereal’ DP is characterized by a higher consumption of buckwheat, other whole grains, cereals, and whole grain pasta and a lower consumption of lard and coconut oil. The consumption of whole grains is a part of healthy DPs Whole grains are a source of dietary fiber, antioxidants, minerals and vitamins, as well as other components such as phytates and phytoestrogens [61]. Currently, no studies have evaluated the impact of whole grain consumption on the cognitive function of young adults. So far, exposure to refined carbohydrates (early in life) has been associated with neurocognitive deficits in later life [62].

Our results showed that the majority of Polish esports players consumed at least three meals a day. Similar results were obtained by Baumann et al. for Norwegian esports students aged 17–21 years, who consumed three to four meals per day [6]. A large group of esports players declared regularity of some meals (47.83%), while irregularity of all eating meals claimed 41.74%. According to Polish nutritional dietary guidelines, 2–3 h intervals between meal consumptions are appropriate [63]. A minority of the esports players (31.30%) consumed their last meal 2–3 h before going to bed, but nearly 68.66% did not follow this dietary guideline. Snacking between meals was also a common feature. Most participants declared that they reached for fruits and sweets when snacking. According to Newzoo’s report, 64% of e-athletes aged 10–65 years in Latin America, North America and Western Europe were regularly consuming salty snacks while playing games, while sweet snacks were followed by soft drinks [64]. Snacking may be caused by irregular eating or low-energy values of meals. In terms of culinary techniques, over 80% of participants declared preparing dishes using frying, and over half chose baking. The participants were asked about the addition of salt to dishes, with around half (47.83%) reporting occasional salting of dishes and sandwiches. The high content of salt in dishes can have a negative impact on blood pressure, which is linked to the risk of cardiovascular diseases [65,66] and, according to actual dietary guidelines, should be limited to 5 g per day [66]. Every second esports player confirmed adding at least two teaspoons of sugar to hot beverages such as tea, coffee or cocoa. Eating excessive amounts of added sugars in a diet, coupled with a sedentary lifestyle, may lead to increased body mass (e.g., body fat) that, in turn, has other health implications. The positive fact is that minimal consumption (1.5–2 L per day) of mineral water was declared by over half participants.

### Limitations

Dietary assessment methods can represent measurement error due to the unreliability of survey participants’ responses. Consequently, we acknowledge that the food and frequency data reported in the present study may be lower or higher than that actually consumed. In addition, our sample was restricted to male esports athletes. Another potential limitation was the lack of information between the reported DPs and tangible health outcomes, such as daily physical activity or more direct measures of cognitive performance reflecting brain function. In subsequent studies, we suggest calculating a coefficient of variation for each participant, based on their specific eating patterns, to more closely examine the relationship between eating patterns and health or cognitive performance. The current results could provide reference data for establishing norms for this population and planning future dietary interventions in this population.

## 5. Conclusions

Nine major DPs were identified among professional and semi-professional Polish esports players. This study found that e-athletes had mostly adherence to unhealthy dietary behaviors and common DPs related to poor dietary quality and high consumption of a high-calorie diet. One identified DP (Vegetable-Fruit) was considered as healthy, but eight DPs were deemed unhealthy and related to a traditional Western diet. Esports players were characterized by a low frequency of consumption of products with potential beneficial effects on brain function, such as fish, nuts, pumpkin seeds, sunflower seeds and other oil seeds. E-athletes were mostly characterized by unhealthy dietary habits that were associated with irregular eating of meals, frequent snacking, at least three meals a day and composition of snacks, the use of frying meat dishes and sweetening hot drinks. More healthy dietary habits included proper hydration during the day and consumption of mainly non-sparkling water. The unhealthy DPs in a group of e-athletes indicate the need to undertake future research assessing knowledge of healthy eating principles and the selection of products that show beneficial effects on brain function, taking into account other factors such as sociodemographics at the time.

## Figures and Tables

**Table 1:** The average consumption frequency (frequency/day) of food items positively influencing brain function.

Food Items	M	SD
Mineral water	5.56	1.65
Hot beverages (tea, coffee, herb or fruit infusions)	4.39	1.63
Spices	4.06	1.63
Table water, spring water	4.03	2.39
Fruits	4.00	1.30
Vegetables	4.00	1.26
Milk (flavored milks, cocoa, coffee with milk)	3.96	1.40
White meat dishes (chicken, turkey, rabbit)	3.68	1.07
Eggs	3.53	1.17
Potatoes (excluding fries and chips)	3.34	1.10
Olive oil and rapeseed oils	3.33	1.43
Fruit juices	3.27	1.41
Fermented milk drinks (yoghurt, kefir)	2.95	1.39
Buckwheats, other whole grains, cereals, whole grain pasta	2.87	1.24
Whole grain bread	2.59	1.53
Cottage cheese (homogenized cheese, cheese deserts)	2.41	1.28
Fishes	2.40	1.10
Nuts, pumpkin seeds, sunflower seeds and other oil seeds	2.38	1.25
Legumes or dishes from legume seeds (beans, peas, lens, soya)	2.12	1.13
Vegetable or vegetable-fruit juices	1.99	1.25
Vegetable canned, marinated or pickled vegetable	1.75	1.08

M—arithmetic mean; SD—standard deviation.

**Table 2:** The average consumption frequency (frequency/day) of food items negatively influencing brain function.

Food Items	M	SD
Light bread (wheat, rye, mixed wheat and rye bread, toast, rolls, rogals)	4.85	1.29
Fried food (meat tor flour)	3.93	1.05
Meats, pork sausages	3.86	1.33
Cheese (molded cheese, blue cheese)	3.67	1.35
White rice, standard pasta, fine groats (manna, cuscus)	3.61	1.04
Sweets (cookies, cakes, chocolate bars, ‘muesli’ types and other confectionery products)	3.52	1.25
Sweet carbonated or non-carbonated beverages (coca-cola, pepsi, sprite, fanta, lemonade)	3.39	1.56
Meat, sausage, poultry and veal	3.21	1.32
Fast food (fries, hamburgers, pizza, chips)	2.88	1.07
Red meat dishes (pork, beef, veal, lamb, venison)	2.73	1.24
Energy drinks (red bull, shot, black horse and others)	2.69	1.44
Margarine or mixes with butter and margarine	2.27	1.54
Lard/coconut oil	2.17	1.42
Alcohol drinks	1.99	1.03
Powder soups or ready soups (can, jar, concentrated) not including frozen soups	1.88	1.10
Canned meat	1.52	0.95

M—arithmetic mean; SD—standard deviation.

**Table 3:** Factor loading matrix for the nine major dietary patterns identified from the Food Frequency Questionnaire (FFQ).

Dietary Patterns (n = 233)									
	Almost Healthy	Western Fast Food	High-Processed Food, Meat and Confectionery	Sweet	Fat-diary Products	Vegetable-Fruit	Spices and Additives	Fats	Cereal
**Variance explained (%)**	9.47	9.22	7.14	5.81	5.56	5.34	4.61	4.08	3.73
**Food and food products**	**Factor loadings**								
White rice, standard pasta, fine groats (manna, cuscus)	**0.663**	−0.001	−0.097	0.040	0.048	0.027	0.056	−0.035	0.107
Whole grain bread	**0.645**	0.133	0.027	−0.062	0.156	−0.035	−0.147	0.066	0.265
White meat dishes (chicken, turkey, rabbit)	**0.644**	0.065	0.293	0.172	−0.049	0.051	0.174	−0.019	−0.187
Fishes	**0.531**	0.344	0.204	−0.073	−0.078	0.049	0.081	0.034	0.127
Nuts, pumpkin seeds, sunflower seeds and other oil seeds	**0.487**	0.213	−0.028	−0.094	−0.092	0.142	0.123	0.313	0.145
Legumes or dishes from legume seeds (beans, peas, lens, soya)	**0.476**	**0.414**	0.136	−0.153	0.150	0.145	0.051	0.092	0.179
Vegetables	**0.435**	0.112	0.130	−0.105	0.017	**0.426**	0.351	−0.069	0.119
Eggs	**0.403**	0.082	0.149	0.002	0.286	−0.007	0.196	0.263	−0.080
Canned meat	0.258	**0.680**	0.103	0.142	0.132	0.006	−0.015	0.101	0.111
Vegetable canned, marinated or pickled vegetable	0.261	**0.679**	0.172	−0.094	0.154	0.028	0.101	0.124	0.071
Vegetable or vegetable-fruit juices	−0.091	**0.662**	0.153	0.012	−0.004	0.316	0.004	0.011	0.190
Powder soups or ready soups (can, jar, concentrated) not including frozen soups	0.106	**0.598**	0.083	0.273	0.137	0.014	−0.099	0.194	−0.011
Fast food (fries, hamburgers, pizza, chips)	0.058	**0.522**	0.099	0.388	−0.156	−0.152	−0.079	−0.048	−0.098
Alcohol drinks	0.097	**0.402**	−0.136	0.152	−0.002	0.007	0.164	0.091	−0.071
Red meat dishes (pork, beef, veal, lamb, venison)	0.355	0.368	0.338	0.122	0.003	0.139	−0.104	−0.108	−0.205
Meats, pork sausages	0.070	0.137	**0.739**	0.060	0.207	0.067	0.072	0.172	−0.038
Meat, sausage, poultry and veal	0.224	0.193	**0.636**	0.043	0.135	0.076	0.141	−0.045	0.073
Light bread (wheat, rye, mixed wheat and rye bread, toast, rolls, rogals)	−0.098	−0.038	**0.621**	−0.067	0.155	0.300	−0.187	0.169	0.073
Fried food (meat tor flour)	0.145	0.182	**0.480**	0.276	−0.004	−0.076	0.152	0.106	0.001
Sweet carbonated or non-carbonated beverages (coca-cola, pepsi, sprite, fanta, lemonade)	−0.163	0.147	0.165	**0.812**	−0.095	0.075	0.074	0.128	−0.070
Energy drinks (red bull, shot, black horse and others)	−0.034	0.250	−0.088	**0.774**	0.179	0.012	−0.090	0.021	0.110
Sweets (cookies, cakes, chocolate bars, ‘muesli’ types and other confectionery products)	0.150	−0.041	**0.409**	**0.503**	0.053	0.066	0.035	−0.024	0.030
Milk (flavored milks, cocoa, coffee with milk)	−0.016	−0.068	0.130	0.036	**0.751**	0.054	−0.009	0.097	0.056
Fermented milk drinks (yoghurt, kefir)	0.182	0.260	0.099	0.003	**0.661**	0.208	0.069	−0.228	0.045
Cheese (molded cheese, blue cheese)	−0.081	0.185	0.335	0.074	**0.515**	0.077	0.216	0.183	0.098
Cottage cheese (homogenized cheese, cheese deserts)	**0.461**	0.316	0.159	−0.007	**0.468**	0.010	0.068	0.105	−0.013
Hot beverages (tea, coffee, herb or fruit infusions)	0.184	−0.274	−0.141	0.174	0.374	**0.660**	0.100	0.256	−0.167
Fruit juices	−0.143	0.367	0.200	0.214	0.055	**0.626**	0.145	0.042	0.043
Fruits	0.374	0.103	0.225	−0.187	0.051	**0.608**	0.089	−0.095	0.208
Potatoes (excluding fries and chips)	0.063	0.094	0.211	0.057	0.186	0.348	0.075	0.295	0.219
Spices	0.002	0.010	−0.026	0.001	0.121	0.226	**0.864**	−0.067	−0.097
Olive oil and rapeseed oils	0.345	−0.005	0.219	−0.004	0.045	−0.005	**0.615**	0.121	0.086
Margarine or mixes with butter and margarine	0.017	0.289	0.271	0.063	0.048	0.142	−0.126	**0.758**	−0.132
Lard/coconut oil	0.249	0.168	0.057	0.207	0.064	−0.122	0.219	**0.534**	**0.429**
Buckwheats, other whole grains, cereals, whole grain pasta	0.268	0.096	0.028	0.019	0.083	0.172	−0.067	−0.029	**0.822**
Mineral water	0.053	−0.029	0.068	−0.073	0.019	0.016	−0.021	0.018	0.035
Table water, spring water	−0.011	0.116	0.123	−0.059	−0.015	0.011	0.088	0.002	0.057

Factor loadings with absolute values > 0.40 are in bold; Method of extracting factors—main components; Rotation method—*Varimax* with Kaiser normalization.

## Data Availability

Not applicable.

## Appendix A

**Table A1:** Esports players dietary habits—percentage response distribution.

Dietary Habits	Responses	Percent
Number of meals	*1 meal*	0.00
	*2 meals*	13.91
	*3 meals*	40.87
	*4 meals*	33.91
	*5 meals and more*	11.30
The frequency of eating between meals	*Never*	5.22
	*1–3 times a month*	6.09
	*Once a week*	13.04
	*Several times a week*	36.52
	*Once a day*	22.61
	*Several times a day*	16.52
The regularity of eating meals	*No*	41.74
	*Yes, but only some*	47.83
	*Yes, all*	10.43
Eating the last meal before going to bed	*2–3 h before bedtime*	31.30
	*Less than 2 h*	11.30
	*There is no rule*	57.39
The composition of the snacks consumed	*Fruits*	41.74
	*Vegetables*	9.57
	*Unsweetened milk drinks and desserts, e.g., yoghurts, cottage cheese, milk*	18.26
	*Sweetened milk drinks and desserts (homogenized cheese, sweetened milk drinks, flavored milk)*	21.74
	*Sweet snacks, e.g., candies, cookies, cakes, chocolate bars, muesli bars, wafers*	39.13
	*Salty snacks, e.g., crackers, sticks, chips, chips*	28.70
	*Nuts, almonds, seeds, pips*	12.17
	*Other products*	9.57
Use of fast-food products (instant soups, ready-made sauces, ready meals, etc.)	*Never*	26.09
	*Almost every day*	5.22
	*Often, i.e., several times a week*	8.70
	*Sometimes, i.e., 5 or more times a month*	33.91
	*Rarely, i.e., 3–4 times a month*	26.09
The type of milk and milk drinks consumed	*Standard fat (full fat)*	66.09
	*Low fat*	27.83
	*Fat free*	0.87
	*I don’t eat dairy products*	5.22
Culinary techniques used to prepare meat dishes	*Boiled*	45.22
	*Braised*	15.65
	*Grilled*	30.43
	*Baked*	53.04
	*Fried*	80.87
	*I don’t eat meat*	0.87
The type of fat used to fry food	*I do not use any frying fat*	6.96
	*I use various fats*	19.13
	*I use vegetable oil (including olive oil)*	47.83
	*I use a margarine*	3.48
	*I use a butter*	20.00
	*I use lard*	2.61
The use of fat as a spread	*I do not use any fat to spread my bread*	21.55
	*I use various fats*	9.48
	*I use a mayonnaise*	1.72
	*I use a margarine*	5.17
	*I use a butter*	56.90
	*I use a mix butter with margarine*	2.59
	*I use lard*	2.59
Sweetening hot drinks (e.g., tea, coffee, cocoa)	*No*	20.87
	*Yes, I sweeten it with one teaspoon of sugar (or honey)*	22.61
	*Yes, I sweeten it with two or more teaspoons of sugar (or honey)*	52.17
	*Yes, I use sweeteners (low-energy sweeteners)*	4.35
The use of salt in dishes and sandwiches at the table	*No*	47.83
	*Yes, but only sometimes*	40.00
	*Yes, I add salt in most of the dishes*	12.17
Daily fluid intake	*Less than 1 L (less than 4 glasses)*	0.00
	*About 1.5 L (about 6 glasses)*	26.96
	*About 2 L (about 8 glasses)*	35.65
	*More than 2 L (more than 8 glasses)*	37.39
The type of water drunk	*I don’t drink water*	0.87
	*I drink non-sparkling water*	69.57
	*I drink sparkling water*	24.35
	*I drink flavored water*	5.22

**Table A2:** The frequency of food items consumption positively influences brain function—The distribution of responses.

Food Items	*The Frequency of Consumption of Food Products—the Percentage Distribution of the Response*						
	1	2	3	4	5	6	7
Mineral water	4.29	2.15	5.58	10.73	15.88	21.46	39.91
Hot beverages (tea, coffee, herb or fruit infusions)	6.87	9.44	12.02	17.17	22.32	28.76	3.43
Spices	12.88	3.86	14.16	26.18	23.61	15.45	3.86
Table water, spring water	30.04	4.29	5.58	13.73	10.30	11.59	24.46
Fruits	3.86	8.15	20.60	34.33	19.31	12.45	1.29
Vegetables	4.72	6.87	16.74	39.48	21.03	9.87	1.29
Milk (flavored milks, cocoa, coffee with milk)	6.87	9.01	15.88	31.76	24.03	11.16	1.29
White meat dishes (chicken, turkey, rabbit)	4.72	7.73	21.46	50.64	13.73	0.00	1.72
Eggs	6.44	10.73	27.47	37.77	14.16	3.00	0.43
Potatoes (excluding fries and chips)	7.73	14.59	23.18	45.92	7.73	0.43	0.43
Olive oil and rapeseed oils	43.35	17.17	17.17	18.03	3.43	0.43	0.43
Fruit juices	11.59	18.45	25.75	29.18	6.44	7.30	1.29
Fermented milk drinks (yoghurt, kefir)	18.88	20.17	24.46	24.46	8.58	2.58	0.86
Buckwheats, other whole grains, cereals, whole grain pasta	14.16	26.18	29.61	21.89	5.15	2.58	0.43
Whole grain bread	34.33	20.60	12.88	21.89	5.15	4.72	0.43
Cottage cheese (homogenized cheese, cheese deserts)	29.18	30.04	19.74	14.16	5.58	0.86	0.43
Fishes	24.89	28.76	31.33	12.45	2.15	0.00	0.43
Nuts, pumpkin seeds, sunflower seeds and other oil seeds	27.47	34.76	18.45	12.02	6.44	0.43	0.43
Legumes or dishes from legume seeds (beans, peas, lens, soya)	37.77	30.04	17.60	12.02	2.15	0.43	0.00
Vegetable or vegetable-fruit juices	48.50	24.89	12.45	9.44	2.58	2.15	0.00
Vegetable canned, marinated or pickled vegetable	57.08	21.89	13.30	5.58	1.29	0.43	0.43

Never less than once a month (1), 1–3 times a month (2), once /twice a week (3), a few times a week (4), once a day (5), 2–3 times a day (6), 4–5 times a day (7).

**Table A3:** The frequency of food items consumption negatively influences the brain function—the distribution of responses.

Food Items	*The Frequency of Consumption of Food Products—The Percentage Distribution of the Response*						
	1	2	3	4	5	6	7
Light bread (wheat, rye, mixed wheat and rye bread, toast, rolls, rogals)	1.72	3.86	10.30	17.60	27.47	36.05	3.00
Fried food (meat tor flour)	3.00	5.58	15.88	52.79	17.60	3.86	1.29
Meats, pork sausages	6.44	8.58	17.60	39.48	16.31	10.73	0.86
Cheese (molded cheese, blue cheese)	7.73	11.59	22.32	31.76	18.45	7.73	0.43
White rice, standard pasta, fine groats (manna, cuscus)	2.15	10.30	30.90	42.49	9.44	4.29	0.43
Sweets (cookies, cakes, chocolate bars, ‘muesli’ types and other confectionery products)	6.01	15.45	23.61	37.34	11.16	6.01	0.43
Sweet carbonated or non-carbonated beverages (coca-cola, pepsi, sprite, fanta, lemonade)	11.59	19.74	22.32	26.61	8.15	7.73	3.86
Meat, sausage, poultry and veal	12.45	18.45	23.18	32.62	9.01	3.86	0.43
Fast food (fries, hamburgers, pizza, chips)	6.87	33.48	31.33	23.18	3.43	1.29	0.43
Red meat dishes (pork, beef, veal, lamb, venison)	16.31	31.33	24.03	22.75	3.86	0.43	1.29
Energy drinks (red bull, shot, black horse and others)	25.32	24.03	24.03	15.88	6.01	3.43	1.29
Margarine or mixes with butter and margarine	46.35	19.74	10.30	13.30	5.15	4.72	0.43
Lard/coconut oil	47.64	20.17	9.44	16.31	4.29	1.29	0.86
Alcohol drinks	37.34	37.77	16.31	6.44	1.29	0.86	0.00
Powder soups or ready soups (can, jar, concentrated) not including frozen soups	48.93	28.33	11.59	7.73	3.43	0.00	0.00
Canned meat	68.24	20.60	4.72	4.29	1.72	0.43	0.00

Never/less than once a month (1), 1–3 times a month (2), once /twice a week (3), a few times a week (4), once a day (5), 2–3 times a day (6), 4–5 times a day (7).
